# Death from Nicotine

**Published:** 1868-06

**Authors:** 


Death from Nicotine.—A case of death from nicotine recently occurred at Cohoes, N. Y., under the following circumstances:—The father of a little girl, in an endeavor to "heal a sore on her lip," applied to it the contents of a "rank" pipe stem. The victim was almost immediately seized with the peculiar symptoms of tobacco poisoning, and died a few hours afterwards.

